# Electricity and Its Use in Veterinary Practice1Read before the Michigan State Veterinary Medical Asssociation, at Lansing, February 7, 1899.

**Published:** 1899-07

**Authors:** W. W. Thorburn

**Affiliations:** Lansing, Mich.


### ELECTRICITY AND ITS USE IN VETERINARY PRACTICE.[1]

By W. W. Thorburn, V.S.,

LANSING, MICH.

There is perhaps no branch of science which received earlier attention or has been the subject of more persistent research than electricity in its various forms.

[1] Read before the Michigan State Veterinary Medical Asssociation, at Lansing, February 7, 1899.

It may not be out of place here to give a brief *résumé* of its history and the practical applications made of it in the past before undertaking to show the position it occupies at present and its promises for the future.

It was fully six centuries before the Christian era that Failes, one of the seven sages of Greece, discovered that amber, when rubbed with a dry cloth, developed a peculiar force capable of attracting light bodies, such as chaff, paper, etc., and in consequence he believed it to be possessed of a soul which was nourished by the attracted objects.

During the third century of our era, at least seven hundred years before the introduction of the compass in European seas, Chinese vessels navigated the Indian Ocean with needles pointing to the south. The magnetic needle was brought from China to Italy in 1260, and one traveller asserts that he saw a pilot in the East Indies direct his course by a compass like those now in use in the year 1500.

Irregular or fitful agitations of the needle were first observed in 1750 by Worgenthin, and later, in 1806, by Humboldt, who gave the accompanying phenomena the name of magnetic storms.

In 1730 Dufaye transmitted electricity along a cord of moist pack-thread for 1300 feet. In 1774 LeSage, a Frenchman, at Geneva, transmitted a message over wires.

It was not till the discovery of the electro-magnet that transmission of messages over wires could be practical. In 1848 Matteuci, who had devoted much time to this subject, conclusively proved that electricity and nervous force are not identical.

The application of electricity to the treatment of disease is now occupying the attention of the medical world more universally than at any previous period of its history.

It is to be regretted that the earlier observers who made those brilliant discoveries which now form the base of all the practical inventions that so astonish the public at the present day have left no fuller account of their experience with disease.

Dr. Poores' experience is that electricity is anodyne, stimulant, sedative, caustic, styptic, or cautery. The resistance of the human body is very great from hand to hand; in a man of ordinary height is equal to 750 miles of telegraph wire. The resistance of bodies interposed in a circuit outside the battery varies according to their composition. Cavendish found that the electric fluid met with as much resistance in passing through a column of water one inch long as through an iron wire of the same diameter four hundred million inches long. And that water containing in solution one part of salt conducts one hundred times better than fresh water, and that a saturated solution of salt conducts seven hundred and twenty times better than fresh water. It is estimated that the human body, when the skin is well moistened, owing to the salts it contains, conducts nearly twenty times better than water.

Prof. Ohm, in 1827, discovered the law which bears his name, and is now made the foundation of all electric measurement. It is this: The strength of the current passing through any part of the circuit varies directly as the difference of potential between its elements and inversely as the resistance in the circuit itself.

Beard and Rockwell have clearly illustrated this point in the following manner: Suppose a current of water is passed through an ordinary syringe, the quantity of water that follows through the tube will be directly proportioned to the force with which it was urged forward by the piston. The force would correspond with an electro-motive force. It may be laid down as a general principal that a feeble current used for a short time produces the greatest therapeutical effect. A very powerful current almost always does harm instead of good, and especially so when it is applied for a considerable length of time. Electricity given in such a way as to frighten, excite, or hurt the patient does more harm than good. A current so strong or so long applied as to produce soreness of the muscles, or fatigue, is a mistake. With regard to the particular condition of the patient with which success or failure occurred, and the strength of current applied, we are left in the dark. Its use until very recent years has beeen entirely empirical, since the specialist who has used galvanism only reports gratifying results in the same class of cases as those that employ faradism. We conclude that at present success depends more on the care with which the details of treatment are carried out than upon the form of electricity employed. Electricity is employed as an aid to diagnosis, to distinguish between apparent and real death, between apparent and real disease, to detect the presence and location of bullets in gunshot wounds, and to locate lameness in various forms.

There is reason to believe that the time is not far distant when the different indications for the particular current best suited to various diseased conditions shall be clearly defined, and to Dr. Rockwell is due the honor of first directing the profession of our country to this path of investigation. We need full and complete clinical reports from observant and careful practitioners before satisfactory knowledge can be obtained of the true place electricity should occupy in our medical and surgical arsenal. Frictional electricity, when applied to medical use, is known as Franklinism, in honor of Benjamin Franklin, who was the first in this country to apply electricity to the treatment of diseases. The first authentic cure by means of an electric machine was in 1744, in the practice of M. Kratzenstein. He succeeded in curing a contracted finger in one-quarter of an hour.

Mr. Morgan says that if a strong shock be passed through the diaphragm sudden contraction of muscles acts violently on the air in the lungs as to produce a shout.

A small charge through the spine instantly deprives the person for a moment of muscular power, and he generally falls to the ground; if the shock is very powerful instant death is the result.

The bodies of animals killed by lightning undergo rapid putrefaction; the blood does not coagulate. Prof. Rothenhal states that muscular death does not correspond with general death of the body, but follows at a period varying from thirty minutes to several hours. He found the post-mortem electric excitability disappears more rapidly after death from chronic than acute diseases. Many reports of cases as now made are worthless, in not being sufficiently explicit in reporting cases. These points should be distinctly brought out after describing the diseased condition in the usual manner.

1. The method of applying the current, whether general or local, continuous or interrupted.
2. The kind of current used.
3. Direction of current and location of electrodes.
4. The interval between treatment.
5. The number of treatments.
6. The length of treatments.
7. Power of battery current employed. It will be noticed that nothing has been said about the strength of the current to be used. If the required dose of drugs to be taken by the mouth into the stomach is still an uncertainty, is it anything to be wondered at that the dose of electricity necessary to be administered to any given case cannot be determined with any degree of accuracy? At least no rule that would be infallible could be laid down for the guidance of the profession.

So I think it advisable to leave the dose to be used to individual experience, remembering it is always better to give too small than too large a dose, and how little will cure, not how much the patient will stand. The duration of treatment and the repetition of same will be left an open question.

I will now give some of my experience with electricity in veterinary practice. It is now about ten years since I first began the use of electricity in various forms of lameness, etc. I used electricity for rheumatism, and with good results, with complete recovery from all pain and lameness after the sixth treatment.

About this time my attention was called to a lame horse, which the owner said had been lame for four or five weeks. This horse was owned by A. Manne, of Lansing. I made a careful examination and came to the decision that the soreness was in the shoulder. As the owner was anxious to have the horse in shape to show, as he thought he had a buyer for him, he wanted to treat him in some manner so as not to blister or gum the hair. I told him I would try the application of electricity. He concluded to have me treat him in that way, and accordingly I secured the horse by backing him into a stall and tying him on both sides with straps and applying a twitch, then moistened the shoulder with chloride of sodium solution. I used a fluid-cell faradic battery with hollow metal electrodes, with wet sponges inside. Applied one sponge over the levator humeri, at about the point it passes over the shoulder-joint, and the other over the caput muscles; gave a mild primary current, continuing for about eight or ten minutes, changing the electrodes frequently. After treatment rubbed thoroughly dry and blanketed. I gave the treatments once a day. He had four in all. First two at my infirmary, and I did not think he showed the improvement he should, so I proposed that the next and following treatments be given at his stable, as it was cold weather and owner had a box-stall not more than eight or ten feet from a large stove, used to warm his shop. In order to get the best results from treatment, animals must be kept warm, especially in cold weather. He received the last treatment at noon, and that evening the owner hooked him with another horse and speeded up and down the pavement with no return of the lameness, and was discharged as all right.

Case No. 2 was a stallion that they were trying to convert from a trotter to a pacer, and in so doing he broke the first hobbles they used on him, and in getting stronger ones and putting on he fought them and strained himself through the loins. At the time that I first saw him he had been hurt about ten days or two weeks. The driver thought he was lame in the front feet, but as treatment of them did no good I was called in to examine the case, and, after noting his action both in and out of harness and by manipulation, I decided the trouble was in the loins; but the driver was not satisfied, and said I would have to prove it still further to convince him. I told him I could do that if that was what he wanted, and to bring the horse to my stable and I would convince him as to who was right. He wanted to know how I was going to do it, and I said by the use of electricity. He consented, and brought the horse, and, after securing in the usual way, I proceeded to moisten the loins and under the crupper with chloride of sodium solution, and then applied one electrode to the loins and the other under the tail, changing the electrodes often, with a primary current, at first working the electrode around near the part I believed to be the sore place, using a current so mild as to cause only very slight contraction of the muscles until I passed the sponge over the sore place, when behold, he resented it as much as to say that is as bad as pricking me with needles, and the driver was satisfied as well as the horse that I had located the sore place. I gave this case seven treatments, extending over a period of fourteen days. When discharged he could move along as free as ever, with no indication of having been injured or undergone a course of treatment.

I could cite a great many more cases, but space will not permit. I use electricity to locate as well as to treat lameness. I have the best of results with electricity in the treatment of congestion of the loins, and I find in practice that it is not how much a horse will stand, but how small a dose will cure. I have the best of results with the current just strong enough so the patient will indicate that he feels it. In the treatment of cases of deep-seated lameness I have found better results from the use of electricity than any other form of treatment that I have used.

There is one great advantage in using electricity in cases of muscular lameness. In fine horses that are held for sale or fancy driving you can treat them and remove all soreness without even leaving a trace of any treatment having been applied.

